# Accurate reconstruction of viral quasispecies spectra through improved estimation of strain richness

**DOI:** 10.1186/1471-2105-16-S18-S3

**Published:** 2015-12-09

**Authors:** Duleepa Jayasundara, I Saeed, BC Chang, Sen-Lin Tang, Saman K Halgamuge

**Affiliations:** 1 Optimisation and Pattern Recognition Research Group, Department of Mechanical Engineering, Melbourne School of Engineering, The University of Melbourne, VIC 3010, Parkville, Australia; 2 Yourgene Bioscience, No. 376-5, Fuxing Rd., Shu-Lin District, New Taipei City, Taiwan; 3 Biodiversity Research Center, Academia Sinica, Taipei 11529, Nan-Kang, Taiwan

**Keywords:** viral quasispecies, quasispecies spectrum reconstruction, strain richness, probabilistic estimation

## Abstract

**Background:**

Estimating the number of different species (*richness*) in a mixed microbial population has been a main focus in metagenomic research. Existing methods of species *richness *estimation ride on the assumption that the reads in each assembled contig correspond to only one of the microbial genomes in the population. This assumption and the underlying probabilistic formulations of existing methods are not useful for quasispecies populations where the strains are highly genetically related.

The lack of knowledge on the number of different strains in a quasispecies population is observed to hinder the *precision *of existing Viral Quasispecies Spectrum Reconstruction (QSR) methods due to the uncontrolled reconstruction of a large number of *in silico *false positives. In this work, we formulated a novel probabilistic method for strain richness estimation specifically targeting viral quasispecies. By using this approach we improved our recently proposed spectrum reconstruction pipeline ViQuaS to achieve higher levels of precision in reconstructed quasispecies spectra without compromising the recall rates. We also discuss how one other existing popular QSR method named ShoRAH can be improved using this new approach.

**Results:**

On benchmark data sets, our estimation method provided accurate richness estimates (< 0.2 median estimation error) and improved the precision of ViQuaS by 2%-13% and F-score by 1%-9% without compromising the recall rates. We also demonstrate that our estimation method can be used to improve the precision and F-score of ShoRAH by 0%-7% and 0%-5% respectively.

**Conclusions:**

The proposed probabilistic estimation method can be used to estimate the richness of viral populations with a quasispecies behavior and to improve the accuracy of the quasispecies spectra reconstructed by the existing methods ViQuaS and ShoRAH in the presence of a moderate level of technical sequencing errors.

**Availability:**

http://sourceforge.net/projects/viquas/

## Background

A number of unsupervised Quasispecies Spectrum Reconstruction (QSR) methods such as ShoRAH [1], QuRe [2], PredictHaplo [3], ViSpA [4] and QuasiRecomb [5] are available in literature. Comprehensive reviews on these methods are presented in [6] and [7]. We recently formulated a novel unsupervised method named ViQuaS [8] for QSR and showed that it outperforms aforementioned popularly used methods.

A major observation on the QSR methods ViQuaS and ShoRAH was that the *precision *(fraction of reconstructed strains that are true: equation 8) of reconstructed spectra was less than the *recall *rate (fraction of true strains that are reconstructed: equation 7) owing to the reconstruction of *in silico *false positives. However, we observed in [8] that ViQuaS has the best *recall *rates among the four methods ViQuaS, ShoRAH, PredictHaplo and QuRe. Also, ShoRAH performs at comparable levels with PredictHaplo in terms of *recall*. Furthermore, both QuRe and Predic-tHaplo demonstrate higher *precision *values than the corresponding *recall *values. Therefore, we realize that the *F-score *(the geometric mean of *recall *and *precision*: equation 9) values of the spectra reconstructed by ViQuaS and ShoRAH can be improved by controlling the generation of false positives without compromising the *recall *rates, but such an improvement cannot be achieved in QuRe and PredictHaplo as the spectra generated by them usually contain a lower number of strains than the actual number of strains in the population. Therefore, other algorithmic changes will be needed to improve the *F-score *values of spectra generated by QuRe and PredictHaplo without compromising the *recall *rates. In this paper, we present a novel probabilistic method to estimate the number of strains in a viral quasispecies population and a strategy to improve the *precision *and *F-score *of ViQuaS analysis pipeline without compromising the *recall *rates, by reducing the number of *in silico *false positives using above estimates as input information. We also show that the same strategy can be used to improve the performance of ShoRAH.

The number of different microbial types in a mixed population is termed as *richness*. Thus far we find two popular methods of estimating the *richness *of a mixed microbial population named PHACCS [9] and CatchAll [10]. The input to both methods takes the same form. In fact, the input is the contig spectrum of the metagenome derived from the target mixed microbial population. Both methods rely on probabilistic parameter estimation strategies. A major assumption regarding the input is that the reads in each contig correspond to only one of the microbial genomes in the population [9], [10]. In other words, it is assumed that the different microbes in the population do not comprise of significant common genomic regions. This assumption is acceptable for populations such as soil, lake water, sea water bacterial populations and bacteriophages, but it is unacceptable for quasispecies populations where the viral strains are highly genetically related. Hence, the requirement arises to formulate the *richness *estimation problem for viral quasispecies in an alternative framework.

The main contributions of this work are two fold. 1) We formulate a novel probabilistic method to estimate the strain richness of a viral quasispecies population and 2) we propose a reconfiguration for the recently published pipeline ViQuaS [8], that significantly improves the *precision *of reconstructed quasispecies spectra without compromising the *recall *rate. Furthermore, we discuss how the existing quasispecies spectrum reconstruction method ShoRAH [1] can benefit from the proposed estimation strategy.

## Methods

### Strain richness estimation

In this work we formulate the strain richness estimation problem as a parameter estimation task given a single observation from a discrete probability distribution.

Consider the instance where the biological sample for next-generation sequencing is collected, at which the quasispecies spectrum can be safely assumed as static. Let us assume the following notations to formulate the proposed estimation problem.

• *L *= Length of the genomic segment of the known reference (or the wild type) genome of the quasispecies population we are interested in reconstructing.

• *s *= Number of different strains in the quasispecies population.

• *r_i _*= Number of mutations in the *i^th ^*strain with respect to the reference genome where *i *∈ {1, 2, 3, ..., *s*}.

• *n *= Number of total possible mutations that can occur in a single strain.

• $r=\frac{\overset{s}{\underset{i=1}{\sum }}r_{i}}{s}$ (Average number of mutations per strain.)

• *U *= The random variable defining the number of unique mutations in the population.

It should be noted that two distinct strains can have one or more common mutations, but not all mutations can be common. For the formulation we assume that each strain contains a constant number of mutations with respect to the reference genome. We use the value *r *as the number of mutations in each strain and define the probability mass function (p.m.f.) of *U *under the parameters *s, n *and *r *(*Pr*(*U *= *u*; *s, n, r*)) as in equation 1.

(1) $$Pr\left(U=u;\;s,\;n,\;r\right)=\left\{\begin{array}{c} \begin{array}{c} 1\;\;\;\text{if}\;s=1\;\text{and}\;u=r \end{array}\\ \overset{r}{\underset{x=0}{\sum }}Pr\left(U=u-x;\;s-1,\;n,\;r\right).P_{s}\left(x\right)\\ \text{if}\;s\in \left(1,\;\left(\begin{array}{c} n\\ r \end{array}\right)\right]\;\text{and}\;u\in \left(r,\;min\left(n,\;rs\right)\right]\\ \begin{array}{c} 0\;\;\;\text{otherwise} \end{array} \end{array}\right)$$

where *n, r, u*, $s\in {\mathbb{Z}}^{+}$ and $x\in {\mathbb{N}}_{0}$ and equation 2 defines *P_s _*(*x*).

(2) $$P_{s}\left(x\right)=\left\{\begin{array}{cc} \frac{\left(\begin{array}{c} u\\ r \end{array}\right)-\left(s-1\right)}{\left(\begin{array}{c} n\\ r \end{array}\right)-\left(s-1\right)} & \text{if}\;x=0\\ \frac{\left(\begin{array}{c} u-x\\ r-x \end{array}\right)\cdot \left(\begin{array}{c} n-u+x\\ x \end{array}\right)}{\left(\begin{array}{c} n\\ r \end{array}\right)-\left(s-1\right)} & \text{if}\;x\in \left[1,\;r\right] \end{array}\right)$$

In real world populations, the number of mutations in each strain may not be a fixed value (*r*), but we show in our results that the effect of this variability on the estimation strategy is minimal under practical settings.

Proof

**Case 1 : ***s *= 1

*s *= 1 corresponds to a population where there is only one strain. If the number of mutations in this strain is *r*, all these mutations are unique in the population. Hence, when *s *= 1 the random variable *U *can only take the value *U *= *r*. Therefore,

*Pr*(*U *= *u*; *s, n, r*) = 1 when *s *= 1 and *u *= *r*

*Pr*(*U *= *u*; *s, n, r*) = 0 when *s *= 1 and *u *≠ *r*

**Case 2 : **$s\in \left(1,\;\left(\begin{array}{c} n\\ r \end{array}\right)\right]$**and ***U *≤ *r*

*s *> 1 corresponds to a population where there are more than one strain. If the number of mutations per strain is *r*, the first strain contributes *r *number of unique mutations towards the value of *U *. The second strain cannot have the same *r *mutations that are observed in the first strain. Therefore, the second strain contains at least one mutation which is different from the already observed *r *mutations. Consequently, when *s >*1, *U *takes a value greater than *r*. Also the total number of strains that are possible to be present is $\left(\begin{array}{c} n\\ r \end{array}\right)$.

Therefore,

*Pr*(*U *= *u*; *s, n, r*) = 0 when $s\in \left(1,\;\left(\begin{array}{c} n\\ r \end{array}\right)\right]$ and *u *≤ *r*

**Case 3 : **$s\in \left(1,\;\left(\begin{array}{c} n\\ r \end{array}\right)\right]$**and ***U > min*(*n, rs*)

Under any circumstance, the number of unique mutations cannot be greater than the number of total possible mutations that can occur in a single strain (*n*). Consequently, the number of unique mutations in the entire population cannot grow beyond *n*.

Furthermore, when $s\in \left(1,\;\left(\begin{array}{c} n\\ r \end{array}\right)\right]$, the first strain contributes *r *number of unique mutations towards the value of *U *and the maximum number any subsequent strain can contribute is also *r*. Hence, the value of *U *cannot be higher than *rs*. As a result, when $s\in \left(1,\;\left(\begin{array}{c} n\\ r \end{array}\right)\right]$, the maximum value attainable by *U *is *min*(*n, rs*). Therefore,

*Pr*(*U *= *u*; *s, n, r*) = 0 when $s\in \left(1,\;\left(\begin{array}{c} n\\ r \end{array}\right)\right]$ and *u > min*(*n, rs*)

**Case 4 : **$s\in \left(1,\;\left(\begin{array}{c} n\\ r \end{array}\right)\right]$**and ***U *∈ (*r, min*(*n, rs*)]

Based on *Case 2 *and *Case 3 *we understand that, when $s\in \left(1,\;\left(\begin{array}{c} n\\ r \end{array}\right)\right]$, *U *can get an integer value within the range (*r, min*(*n, rs*)].

Consider the situation where we have observed *U *= *u *- *x *with (*s *- number of strains. The probability of occurrence of this situation is *Pr*(*U *= *u *- *x*; *s *- 1, *n, r*). In order to observe *U *= *u *with *s *number of strains, the remaining *s^th ^*strain should contain exactly *x *number of new unique mutations. *x *can be any natural number in the range [0*, r*]. Hence, the probability of observing *U *= *u *with *s *number of strains is,

$$Pr\left(U=u;\;s,\;n,\;r\right)=\overset{r}{\underset{x=0}{\sum }}Pr\left(U=u-x;\;s-1,\;n,\;r\right).P_{s}\left(x\right)$$

where *P_s _*(*x*) is the probability of observing *x *(*x *∈ [0, *r*]) number of new unique mutations in the *s^th ^*strain.

Consider the case where *x *∈ [1*, r*]. For the *s^th ^*strain to contain exactly *x *number of new unique mutations out of a total of *r*, only (*r - x*) number of mutations in the *s^th ^*strain can be a subset of the already observed (*u - x*) number of unique mutations. The remaining *x *number of mutations should arise from the unobserved *n - *(*u - x*) possible mutations. In addition, the total number of ways that these *r *mutations can occur without being equivalent to one of the (*s - *1) number of already observed ways is $\left(\begin{array}{c} n\\ r \end{array}\right)-\left(s-1\right)$.

Hence, when *x *∈ [1*, r*],

$$P_{s}\left(x\right)=\frac{\left(\begin{array}{c} u-x\\ r-x \end{array}\right)\cdot \left(\begin{array}{c} n-u+x\\ x \end{array}\right)}{\left(\begin{array}{c} n\\ r \end{array}\right)-\left(s-1\right)}$$

Consider the case where *x *= 0. In this case all *r *mutations of the *s^th ^*strain must come from the already observed *u *unique mutations in the (*s *- 1) number of strains. However, the *s^th ^*strain cannot be equivalent to any of the already observed (*s *- 1) strains. The number of ways this scenario can occur is $\left(\begin{array}{c} u\\ r \end{array}\right)-\left(s-1\right)$. Similar to the case where *x ∈ *[1*, r*], the total number of ways that these *r *mutations can occur without being equivalent to one of the (*s - *1) number of already observed ways is $\left(\begin{array}{c} n\\ r \end{array}\right)-\left(s-1\right)$

Hence, when *x *= 0,

$$P_{s}\left(x\right)=\frac{\left(\begin{array}{c} u\\ r \end{array}\right)-\left(s-1\right)}{\left(\begin{array}{c} n\\ r \end{array}\right)-\left(s-1\right)}$$

We observe that for all *U *= *u *where *u *∈ [*r, n*), there exist a global optimum value in the likelihood function of *s *given *U *(i.e. *L*(*s*|*U *= *u*)) such that *s *is finite. Hence, given an observed value for *U *we can readily use the maximum likelihood strategy to estimate the value of *s*. A closed form equation for the maximum likelihood estimator of *s *(*s_mle_*) cannot be derived. Nevertheless, *s_mle _*can be obtained numerically in a time efficient manner using dynamic programming.

The expected value of *U *given the value of *s *(i.e. *E*(*U*|*s*)) is a strictly increasing discrete valued function of *s *for given values of *n *and *r*. Therefore, as a second strategy we can estimate *s *using the method of moments.

In real world quasispecies populations, we can assume that *r, s *<<<*n*. Since it is impractical to know the value of *n*, we set *n *= *L*. This approximation is observed to have minimal effect on the parameter estimators when *r, s *<<<*L *(i.e. when *r, s *<<<*n *the exact value of *n *has minimal effect on the estimated value of *s*).

### Calculating *r *given a NGS metagenome

For a given metagenome the chance of each strain being completely sequenced, decreases with its relative frequency. [11] extends the Lander-Waterman model of sequencing [12] to derive the probability that all the strains are covered by the given number of sequenced reads assuming the reads are uniformly distributed along the genomic region of interest. The theoretically re-constructible minimum relative frequency (*f_min_*), derived according to [11] and [13], defines a relative frequency value for the strains in the population above which the probability of a strain being completely covered is at least *p_min_*. We set *p_min _*= 0.99 in our study.

Given a NGS metagenome, we use ViQuaS algorithm presented in [8] to reconstruct the unsupervised quasispecies spectrum of the viral population. We demonstrated in [8] that ViQuaS reconstructs a highly reliable spectrum for strains having a relative frequency greater than the theoretically re-constructible minimum relative frequency (*f_min_*). Hence we obtain an approximate value for *r *by calculating the median number of mutations present in the reconstructed strains having a relative frequency greater than *f_min_*.

Alternatively, if we can safely assume that the sequenced reads provide a uniform coverage across the genomic region of interest, following formulae can be used to calculate the parameter *r*.

(3) $$coverage=\frac{\begin{array}{c} \text{Total}\;\text{number}\;\text{of}\;\text{sequenced}\;\text{bases}\\ \text{aligned}\;\text{within}\;\text{the}\;\text{genomic}\;\text{region} \end{array}}{\text{length}\;\text{of}\;\text{the}\;\text{genomic}\;\text{region}\;\left(L\;\text{bp}\right)}$$

(4) $$r=\frac{\begin{array}{c} \text{Total}\;\text{number}\;\text{of}\;\text{mutations}\;\text{in}\;\text{reads}\\ \text{aligned}\;\text{within}\;\text{the}\;\text{genomic}\;\text{region} \end{array}}{coverage}$$

### Calculating *U *given a NGS metagenome

Depending on the information we are interested in, we use different strategies to calculate *U *. Let us denote *s_t _*as the total number of strains in the population (i.e. *richness*) and *s_f _*as the number of strains having a relative frequency greater than *f_min_*.

Each read in the metagenome carries zero or more mutations belonging to a single strain of the original population. However, it is unlikely that all the mutations carried by the strains having a relative frequency less than *f_min _*are captured during sequencing. Hence, defining *U *as the number of unique mutations captured in all reads of the metagenome leads us to an estimate of *s $\left({ŝ}_{t}\right)$*less than *s_t _*(i.e. a lower bound for *s_t_*).

As a second strategy, we define *U *as the number of unique mutations captured in the *local haplotypes *of the ViQuaS analysis pipeline having a local haplotype frequency greater than *f_min_*. Due to the presence of common genomic regions, reads originating from two or more low frequency strains can form a single *local haplotype *having a local haplotype frequency greater than *f_min_*. Therefore, the second strategy gives us a *U *value corresponding to a number of strains slightly higher than *s_f_*. Hence, the estimated value $\left({ŝ}_{f}\right)$ is an upper bound for *s_f_*.

### Reconfiguration of ViQuaS

The ViQuaS analysis pipeline presented in [8] performs an unsupervised reconstruction of strains given a NGS read set. We propose to reconfigure the original ViQuaS pipeline as follows. First, the unsupervised algorithm provides information needed to calculate the parameter *r *and the observation *U *as outlined in the previous subsections, facilitating the calculation of ${ŝ}_{f}$. This ${ŝ}_{f}$ value is fed back to the global spectrum reconstruction algorithm of ViQuaS to decide whether it should continue with the unsupervised result or terminate after having reconstructed ${ŝ}_{f}$ number of strains. Assume the unsupervised ViQuaS pipeline has reconstructed *s_f,u _*number of strains with a frequency greater than *f_min_*. Then,

if, $s_{f,u}>{ŝ}_{f}\Rightarrow$ Terminate after ${ŝ}_{f}$ strains else, continue with the unsupervised result

### Data sets

For the ease of comprehension and comparison of results, we used the simulated data sets (*SS1, SS2, SS3, SS4, SS5, SS6, SS7 *and *SS8 *) described in detail in [8] to benchmark the ViQuaS and ShoRAH using the proposed reconfigurations presented in this paper. (We have provided the description in [8] as Additional File 1 for the ease of comprehension.) We also used the V11909 real Roche 454 HIV-1 data set to demonstrate the applicability of the estimation strategy on real data. Details of V11909 are provided in [8].

## Results

### Validation of strain richness estimation theory

We used 4800 simulated quasispecies populations with known input parameters to validate the strain richness estimation theory for practical settings where the number of mutations per strain and the number of strains are much less than the target genome length (*r, s *<<<*L*). The simulated samples have the following parameter spaces: *n *= 1000, *r *∈ {5, 10, 15, 20, 25, 30, 35, 40, 45, 50} and *s *∈ {3, 5, 7, 10, 25, 50, 75, 100}. Apart from the three parameters mentioned in *Strain richness estimation *subsection, we used the parameter *v *∈ {0, 0.1, 0.2, 0.3, 0.4, 0.5} to model the variability of *r *within a given population. For example, each simulated population has *s *number of strains and the distribution of the number of mutations in each strain has a discrete normal distribution with mean *r *and standard deviation *vr*.

**Figure 1:**
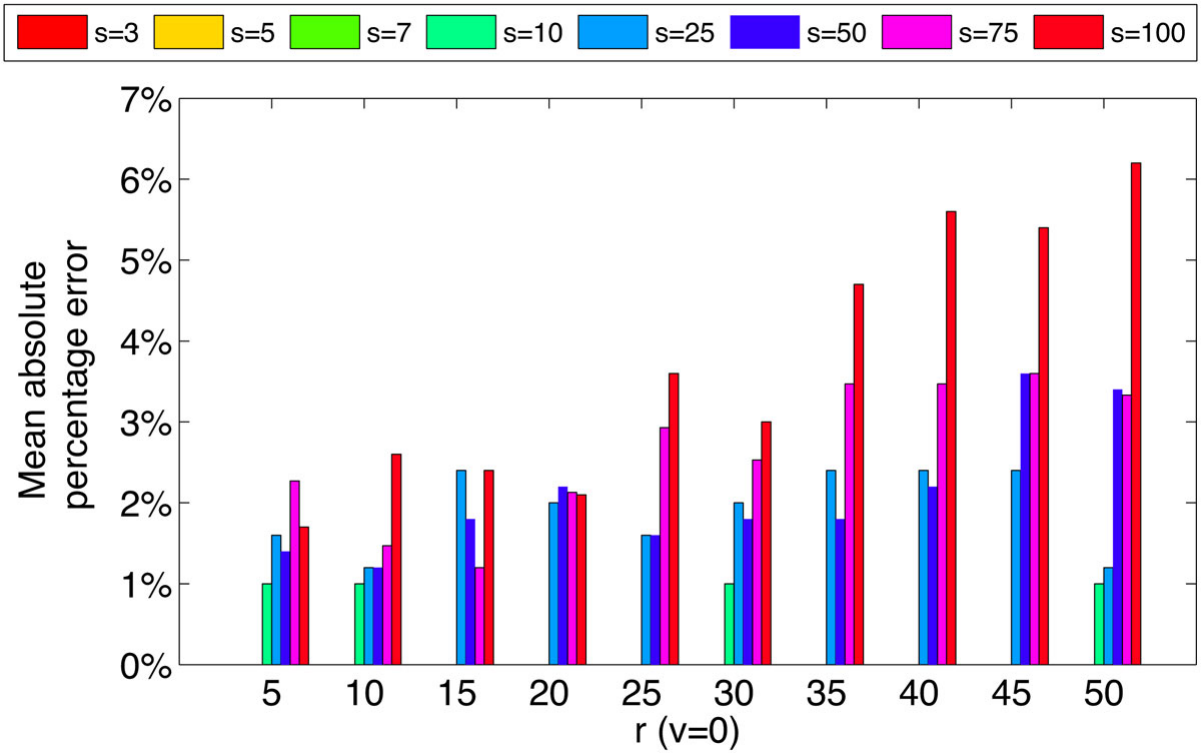
**Mean absolute percentage error of parameter *s *when *v *= 0 and *n *= 1000 for different values of *r *and *s***.

Figure 1 summarizes the performance of Method of Moments (MoM) estimation strategy for the parameter *s *under different values of *r *and *v *= 0 (i.e. constant number of mutations per strain). For the simulated ranges of *r *and *s*, estimation error shows a roughly increasing pattern with both *r *and *s*, while keeping the mean error below 0.07. We observed that the estimated values are highly accurate (mean absolute percentage error < 1%) when the number of strains (*s*) is small (i.e. *s *= 3, 5, 7, 10) but the estimation error grows high with the value of *s *when *s *> 10 for our simulated samples. Equation 5 was used to calculate absolute percentage error:

(5) $$\text{absolute}\;\text{percentage}\;\text{error}=\frac{|\text{estimated}\;\text{value}-\text{true}\;\text{value}|*100\%}{\text{true}\;\text{value}}$$

**Figure 2:**
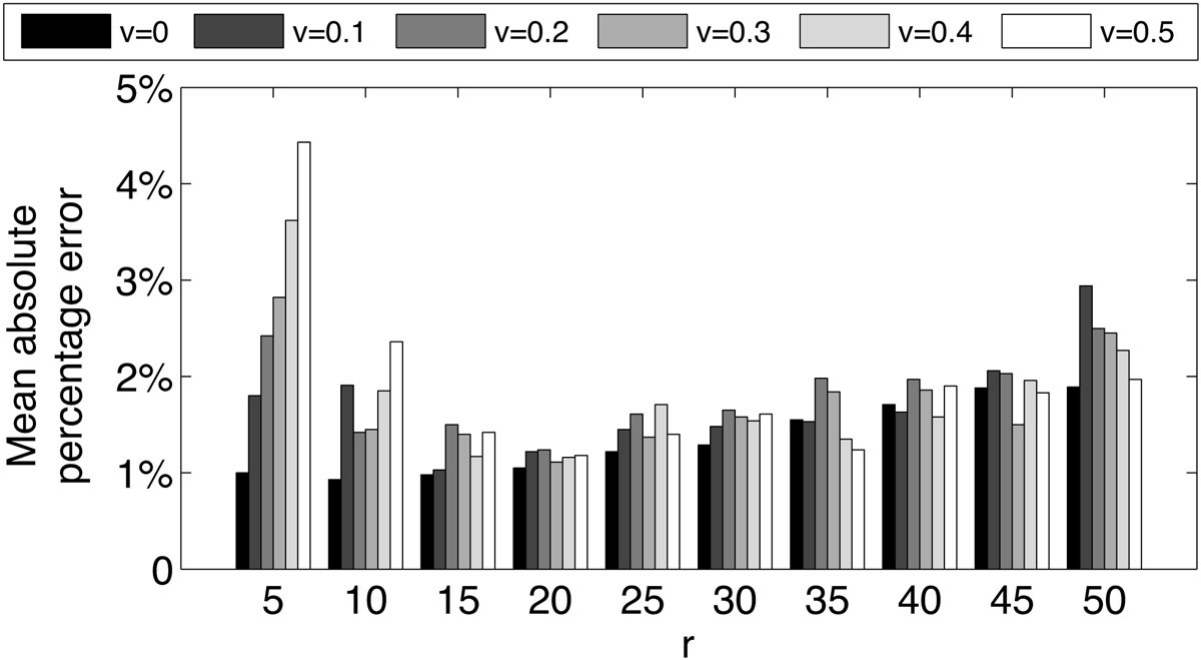
**Mean absolute percentage estimation error of parameter *s *when *n *= 1000 for different values of *r *and *v *averaged over all values of *s***.

Figure 2 shows the variations of estimation error of *s *when the number of mutations per strain is variable with a mean value of *r *and a standard deviation of *vr*. Estimates are highly sensitive to the variability in the number of mutations per strain when the mean is small (i.e. when the strains are highly similar to each other or the populations are less diverse), but shows minimal sensitivity when the mean values increase (i.e. when the populations are considerably diverse), while keeping the mean error over all values of *s *below 0.05. These two observations (Figures 1 and 2 validate our estimation strategy as well as the simplification used in our calculations to consider *r *as a fixed parameter for a given population ignoring the variability in the number of mutations per strain.

### Estimating *s_t _*and *s_f _*in quasispecies populations using NGS data

**Figure 3:**
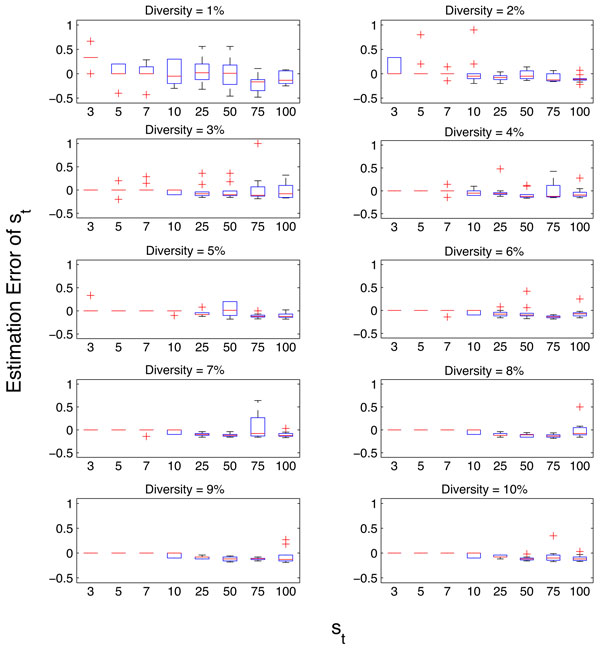
**Estimation error of *s_t _*in quasispecies populations having different *Diversity *and *s_t _*values of simulated sample set *SS*1**.

**Figure 4:**
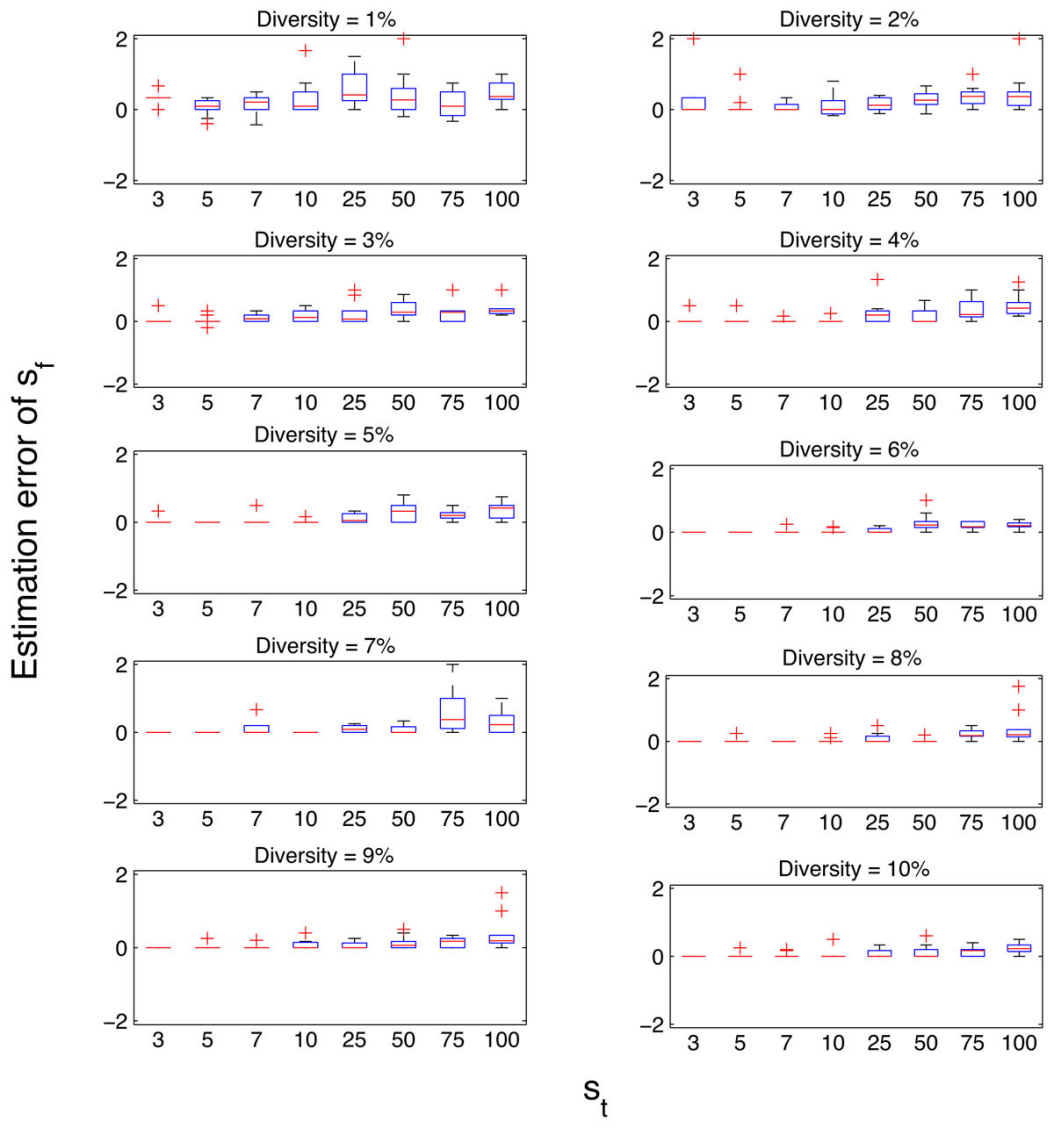
**Estimation error of *s_f _*in quasispecies populations having different *Diversity *and *s_t _*values of simulated sample set *SS*1**.

We used the simulated data set *SS1 *to evaluate the performance of the richness estimation method on NGS data derived from viral quasispecies populations. For each sample, the value of the random variable *U *was calculated at two stages as described under the *Calculating U given a NGS metagenome *subsection corresponding to *st *and *s_f_*. Figures 3 and 4 illustrate the distributions of estimation errors of *s_t _*and *s_f _*respectively. Estimation error was calculated as follows:

(6) $$\text{estimation error}=\frac{\text{estimated value}-\text{true value}}{\text{true value}}$$

We observed that the estimation errors of both parameters decrease with increasing *Diversity *(*Diversity *∝ *r*) and increase with increasing *s_t_*. Furthermore, we observe that the estimation errors of *s_t _*are predominantly negative (706 out of 800 instances in *SS1 *) and that of *s_f _*are predominantly positive (787 out of 800 instances in *SS1 *) and the magnitudes of median error are close to zero. The major causes of positive estimation error of *st *are: (i) significant difference between the calculated *r *and the actual *r *used in simulating data and (ii) the existence of samples where $s_{t}≃s_{f}$. The major cause of negative estimation error of *s_t _*is the significant difference between the calculated *r *and the actual value. This confirms our claim in *Calculating U given a NGS metagenome *subsection that the corresponding estimates (${ŝ}_{t}$ and ${ŝ}_{f}$) give a lower bound for *s_t _*and an upper bound for *s_f_*.

### Enhanced QSR performance in ViQuaS and ShoRAH

**Figure 5:**
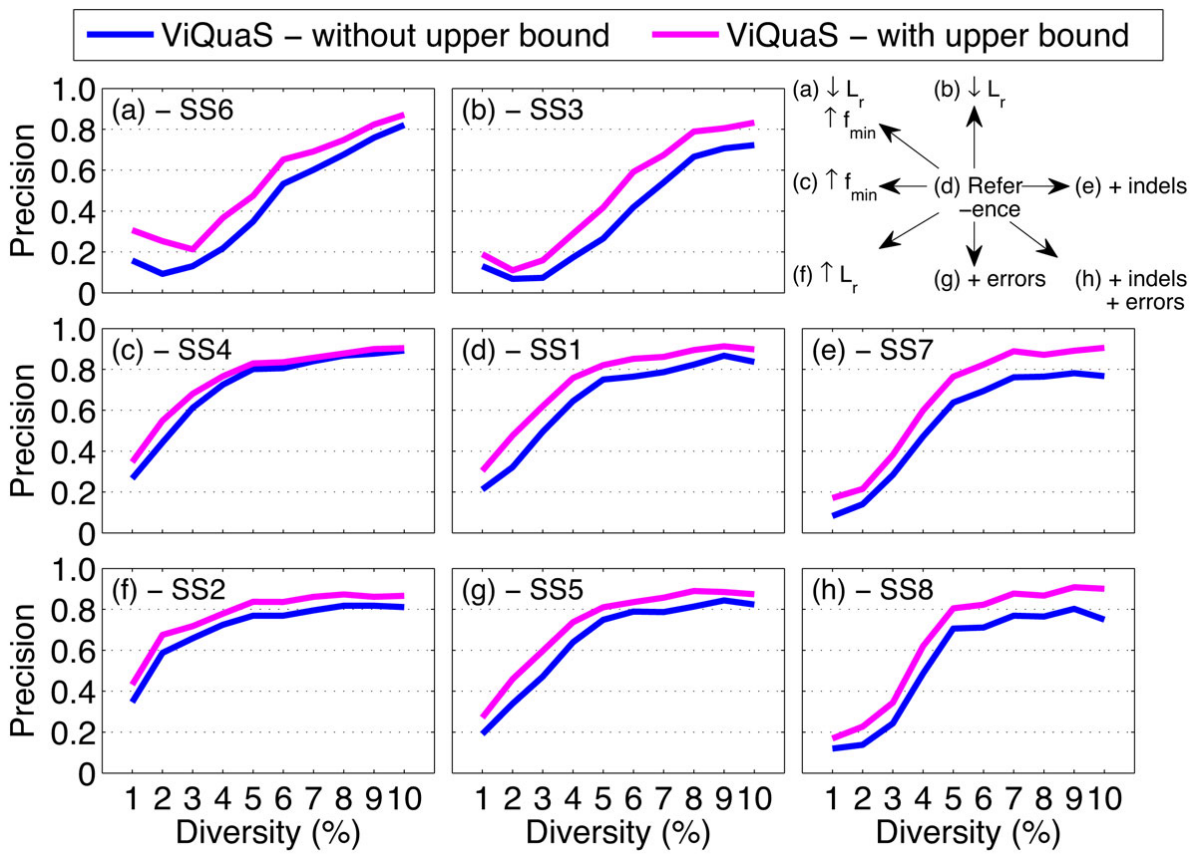
**Performance comparison of ViQuaS in terms of precision with and without using the estimated upper bound for *s_f_***. Each point indicates mean value of the measure.

**Figure 6:**
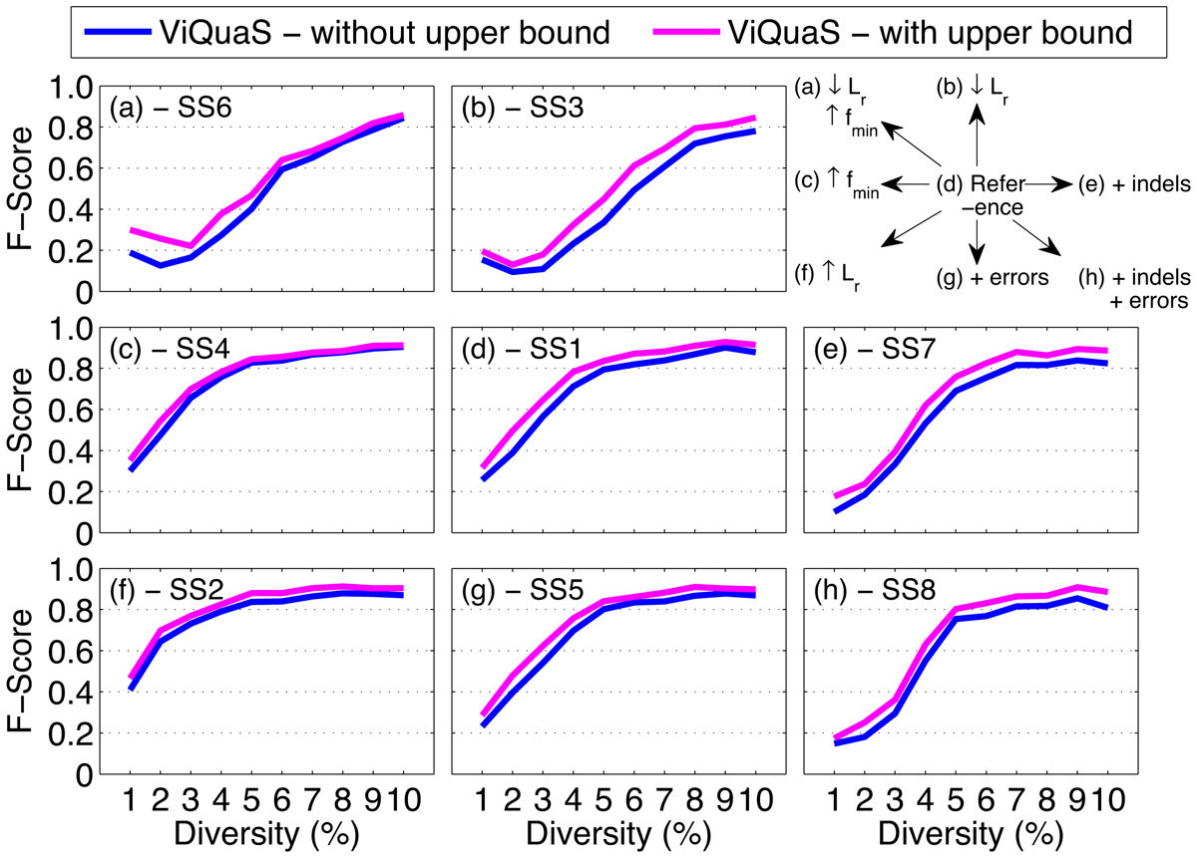
**Performance comparison of ViQuaS in terms of F-score with and without using the estimated upper bound for *s_f_***. Each point indicates mean value of the measure.

In our previous work [8] we observed that the precision of reconstructed quasispecies spectra are significantly lower than the recall rate under all simulation settings due to the reconstruction of a significantly higher number of false positive strains. Using the estimated value ${ŝ}_{f}$ as an upper bound for *s_f _*we controlled the growth of false positive strains and obtained significant gain in *precision *(2%-13%) as shown in Figure 5. Consequently we also observed significant gain in *F-score *values (1%-9%) for reconstructed spectra using ViQuaS (Figure 6). (Refer [8] for the comparison of performance between the methods ViQuaS, ShoRAH, QuRe and PredictHaplo under the same simulation settings.)

Furthermore, we applied the same reconfiguration we proposed on Vi-QuaS using the estimated upper bounds for *s_f _*$\left({ŝ}_{f}\right)$ on an existing QSR methods named ShoRAH to evaluate the performance enhancement attainable via our new estimation strategy. Table 1 summarizes the gain in *precision *and *F-score *attained by the two methods, ViQuaS and ShoRAH. Similar to ViQuaS, we observe that ShoRAH also attains considerable gain in *precision *and *F-score *when the added knowledge of ${ŝ}_{f}$ values is used. Most importantly, no compromise in *recall *rates were observed associated with the gain in *precision *of both ViQuaS and ShoRAH. (Original recall values are presented in [8]. Performance measures of QuRe and Predic-tHaplo are not included in this table as they cannot be improved using the proposed reconfiguration strategy.) The three performance measurement terms *recall, precision *and *F-score *were calculated according to the following equations. Further details regarding the calculations are found in [8].

**Table 1:** Comparison of the improvement in ViQuaS and ShoRAH with and without using the estimated upper bound for *s_f _*$\left({ŝ}_{f}\right)$ under different quasispecies population characteristics and NGS sequencing characteristics when *Diversity *> 3%

Sample Set Name	Method	*Precision *without ${ŝ}_{f}$	*Precision *with ${ŝ}_{f}$	*F-score *without ${ŝ}_{f}$	*F-score *With ${ŝ}_{f}$
*SS1*	ViQuaS	**0.782**	**0.857**	**0.830**	**0.875**
	ShoRAH	0.657	0.710	0.682	0.717

*SS2*	ViQuaS	0.786	**0.845**	**0.851**	**0.887**
	ShoRAH	**0.791**	0.809	0.805	0.816

*SS3*	ViQuaS	**0.499**	**0.628**	**0.560**	**0.647**
	ShoRAH	0.381	0.442	0.403	0.445

*SS4*	ViQuaS	**0.830**	**0.853**	**0.853**	**0.867**
	ShoRAH	0.776	0.815	0.778	0.803

*SS5*	ViQuaS	**0.778**	**0.841**	**0.827**	**0.865**
	ShoRAH	0.664	0.718	0.687	0.723

*SS6*	ViQuaS	**0.566**	**0.661**	**0.594**	**0.656**
	ShoRAH	0.405	0.473	0.420	0.468

*SS7*	ViQuaS	**0.765**	**0.821**	**0.786**	**0.818**
	ShoRAH	0.000	0.000	0.000	0.000

*SS8*	ViQuaS	**0.776**	**0.829**	**0.796**	**0.827**
	ShoRAH	0.000	0.000	0.000	0.000

(7) $$Recall=\frac{\text{True}\;\text{Positive}\;\text{Strains}\;\text{with}\;\text{a}\;\text{relative}\;\text{frequency}>f_{min}}{\text{Expected}\;\text{Number}\;\text{of}\;\text{Strains }\left(N_{p}\right)}$$

(8) $$Precision=\frac{\text{True}\;\text{Positive}\;\text{Strains}\;\text{with}\;\text{a}\;\text{relative}\;\text{frequency}>f_{min}}{\text{Total}\;\text{number}\;\text{of}\;\text{reconstructed}\;\text{strains}\;\text{with}\;\text{a}\;\text{relative}\;\text{frequency}>f_{min}}$$

(9) $$F\text{ - }Score=2\times \frac{\left(Recall\times Precision\right)}{\left(Recall+Precision\right)}$$

### Application on real data

Analyzing V11909 [14] using the estimation method we found that it contains 16 mutations per strain on average (*r *= 16) within the 1044 bp long region of interest. The *Diversity*, number of strains, exact nucleotide sequences and the relative frequencies of the strains in the population are unknown. The estimated technical error rate of this sample is 0.11% [13], mean read length is 90bp and the total number of reads (*n_total_*) is 5177. The theoretically re-constructible minimum relative frequency (*f_min_*) for this set of reads is 2.5%. The number of unique mutations found in the total set of reads was 752 and the number of unique mutations found in the local haplotypes having a relative frequency greater than *f_min _*was 90. Accordingly, our method estimated that ${ŝ}_{t}=82$ and ${ŝ}_{f}=6$ for V11909 HIV-1 data set using the p.d.f. *Pr*(*U*; *s, n *= 1044, *r *= 16).

## Discussion and Conclusions

To the best of our knowledge there exists no dedicated method in literature to estimate the number of strains in a viral quasispecies population. Our previous studies highlighted that the unsupervised quasispecies spectrum reconstruction methods such as ShoRAH [1] and QuRe [2] reconstruct respectively higher and lower number of *in silico *false positive strains. These methods were not aimed at providing accurate estimates for the number of strains in a population. Estimation of species richness in mixed microbial populations is a problem that is closely related to the problem discussed here. However, methods addressing mixed microbial population richness estimation are not applicable to the problem at hand due to the characteristic differences between the subjective populations. We presented in this paper a novel probabilistic method to estimate the number of strains in a quasispecies population based on the distribution of mutations among different strains.

The derived p.m.f. *Pr*(*U *= *u*; *s, n, r*) shows a significant relationship to the well known hypergeometric distribution. We were unable to derive closed form expressions for the maximum likelihood and Method of Moments estimators for the parameter *s*. They were calculated using dynamic programming as both estimators are deterministic. We chose to use the Method of Moments estimator as it provided marginally better estimates than the maximum likelihood estimator on benchmark data sets.

We demonstrated that the variability in the number of mutations per strains in a population has minimal effect on the estimation method under practical parameter values of *n, r *and *s*. However, the estimates are considerably sensitive to the parameter *r*. This implies that identifying the location of the distribution (i.e. median, mean or mode) of the number of mutations per strain is a critical step in the presented method. Accordingly, we identified that significant differences between the calculated and correct *r *values as the main cause of the outliers (high error values) of the error plots in Figures 3 and 4.

Using the estimated upper bound of *s_f _*$\left({ŝ}_{f}\right)$, we reconfigured the Vi-QuaS analysis pipeline to control the growth of *in silico *false positives. The summarized results presented in Figures 5, 6 andTable 1 show that the reconfigured ViQuaS pipeline improves the *precision *and *F-score *of reconstructed spectra compared to the previously proposed ViQuaS pipeline [8]. The highlight of the proposed reconfiguration is that we use the knowledge from both the unsupervised algorithm and the probabilistic estimation method to make a well informed decision to limit the number of false positives. This strategy allows the reconfigured ViQuaS pipeline to compensate for errors introduced by: (i) the uncontrolled growth of strains when using the unsupervised algorithm alone and (ii) the sensitivity of the estimation method to calculation errors of the parameter *r*.

We also demonstrated that, similar to ViQuaS, the performance of the existing method ShoRAH can be substantially improved using the added knowledge of the estimated ${ŝ}_{f}$ values. It will be an interesting study to see whether QuRe and PredictHaplo can be improved by giving the estimated ${ŝ}_{f}$ values as input to the global spectrum reconstruction stage of the algorithms. This will require the ability to edit the source code of the respective methods. Also, the reconfiguration proposed in this paper can be used to reduce the computational cost to a certain extent by appropriately reducing the number of strains being reconstructed in the global strain reconstruction stage of a quasispecies spectrum reconstruction pipeline.

Although the method can handle an error rate of 0.1% (theoretical maximum substitutional error probability of quality trimmed reads with a PHRED threshold of 30), we identify the absence of a proper discounting method to overcome the effect of technical sequencing errors (in cases of very high error rates) when calculating different parameters of the estimation method as the main drawback of our proposal. We plan to study further on such a discounting strategy that can overcome both substitutional and homopolymeric errors.

We also plan to study further on the p.m.f. with the view of improving the efficiency of the calculation methods and providing sound mathematical proof for the observed properties of the parameter estimators. We hope this new probability distribution will be useful in many theoretical and applied statistical problems in the future.

In addition to modeling the distribution of mutations as proposed in this paper, a quasispecies population may also be modeled using master equations [15-17] to estimate the strain richness. However, solving highly complex master equations has been a main bottleneck for decades [18] when using such techniques.

## Competing interests

The authors declare that they have no competing interests.

## Authors' contributions

DJ formulated the problem and the statistical solution, carried out the simulations, analyzed real data and wrote the manuscript. IS and SKH contributed in formulating the statistical solution, designing the validation and simulation studies and preparing the manuscript. BCC and SLT contributed in designing the simulation study, analyzing real data and preparing the manuscript.

## Supplementary Material

Additional File 1 **Description of simulated data sets**. Detailed description of the simulated data sets *SS1*-*SS8*. Click here for file

## Declarations

Publication charges for this article have been funded by The University of Melbourne.

This article has been published as part of *BMC Bioinformatics *Volume 16 Supplement 18, 2015: Joint 26th Genome Informatics Workshop and 14th International Conference on Bioinformatics: Bioinformatics. The full contents of the supplement are available online at http://www.biomedcentral.com/bmcbioinformatics/supplements/16/S18.
