# Supplementary material for: Accurate reconstruction of viral quasispecies spectra through improved estimation of strain richness
Source: BMC Bioinformatics. 2015 Dec 9;16(Suppl 18):S3. doi: 10.1186/1471-2105-16-S18-S3 (PMC4682401; doi:10.1186/1471-2105-16-S18-S3)
Supplement: Additional File 1 — Description of simulated data sets. Detailed description of the simulated data sets SS1-SS8. [file 1471-2105-16-S18-S3-S1.pdf]

Supplementary File 1 - Description of simulated data sets

We used the software named Grinder to simulate reads with a Hepatitis B genome (NCBI accession number NC_003977) as the reference (G_{ref}). All three types of mutations (T) (SNPs ($T = P$), insertions ($T = I$) and deletions ($T = D$)) were simulated in the range from 1814 to 2813 nucleotide positions of the selected reference genome where insertions and deletions have lengths ranging from 1bp to 3bp (a $1000bp$ range that includes the *pre-C/core* gene of Hepatitis B was selected in order to evaluate the reconstruction capability on a genomic region much larger than the mean read length used). Samples with three different read length distributions were generated with parameters $\mathcal{N}(L_r, 25)$ where $L_r \in \{100bp, 200bp, 300bp\}$. The coverage depth was set to be uniform throughout the total genome length.

The simulated samples were categorized into 8 different sets, each consisting of 800 samples, according to their population characteristics ($Diversity, N_s, T$) and NGS read characteristics (L_r, n_{total}, e) as shown in Table S2 of Supplementary File 4. At each of the 80 $Diversity$ - N_s value pair configurations, 10 samples were generated with different relative frequencies, resulting 800 samples per each sample set. In order to keep the analysis as unbiased as possible, the relative frequencies of strains were randomly chosen from the range 0.001% - 99.999% without restricting to any theoretical distribution.

The relative frequency distributions and simulated mutations were kept the same across samples in *SS1*, *SS2*, *SS3*, *SS4*, *SS5*, and *SS6* as well as across samples in *SS7* and *SS8*. For example, the 5th sample in both *SS7* and *SS8* has equal $Diversity$, N_s, L_r, n_{total}, the same set of strains and the same relative frequency distributions while only differing in e values of the two samples.

Table S1: Simulated sample set categorization: *SS1* - *SS8*

Sample Set Name	$Diversity(\%)$	N_s	T	$L_r(bp)$	n_{total}	$e(\%)$	$f_{min}(\%)$
SS1	1 - 10	3 - 100	P	200	30000	0	0.7
SS2	1 - 10	3 - 100	P	300	20000	0	0.7
SS3	1 - 10	3 - 100	P	100	60000	0	0.7
SS4	1 - 10	3 - 100	P	200	10000	0	2.1
SS5	1 - 10	3 - 100	P	200	30000	0.1	0.7
SS6	1 - 10	3 - 100	P	100	30000	0	1.4
SS7	1 - 10	3 - 100	P, I, D	200	30000	0	0.7
SS8	1 - 10	3 - 100	P, I, D	200	30000	0.1	0.7

$Diversity$ is the average hamming distance between all strains in the quasispecies population ($Diversity \in \{1\%, 2\%, 3\%, 4\%, 5\%, 6\%, 7\%, 8\%, 9\%, 10\%\}$), N_s is the total number of strains in the quasispecies population ($N_s \in \{3, 5, 7, 10, 25, 50, 75, 100\}$), T is the types of mutations present in the population ($T = P$ for SNPs, $T = I$ for Insertions and $T = D$ for Deletions), L_r is the mean read length (read length $\sim \mathcal{N}(L_r, 25)$), n_{total} is the total number of reads in one sample, e is the substitutional error probability of the reads and f_{min} is the theoretically reconstructible minimum relative frequency.
